# The emerging roles of PHOSPHO1 and its regulated phospholipid homeostasis in metabolic disorders

**DOI:** 10.3389/fphys.2022.935195

**Published:** 2022-07-26

**Authors:** Yi Liu, Yingting Wu, Mengxi Jiang

**Affiliations:** ^1^ Department of Pharmacology, School of Basic Medical Sciences, Capital Medical University, Beijing, China; ^2^ Advanced Innovation Center for Human Brain Protection, Capital Medical University, Beijing, China

**Keywords:** PHOSPHO1, phospholipid homeostasis, metabolic disorders, mitochondrial function, lipid droplets

## Abstract

Emerging evidence suggests that phosphoethanolamine/phosphocholine phosphatase 1 (PHOSPHO1), a specific phosphoethanolamine and phosphocholine phosphatase, is involved in energy metabolism. In this review, we describe the structure and regulation of PHOSPHO1, as well as current knowledge about the role of PHOSPHO1 and its related phospholipid metabolites in regulating energy metabolism. We also examine mechanistic evidence of PHOSPHO1- and phospholipid-mediated regulation of mitochondrial and lipid droplets functions in the context of metabolic homeostasis, which could be potentially targeted for treating metabolic disorders.

## Introduction

Phosphoethanolamine/phosphocholine phosphatase 1(PHOSPHO1) hydrolyzes phosphocholine to choline and phosphate (Pi) or hydrolyzes phosphoethanolamine to ethanolamine and Pi (Roberts et al., 2004). PHOSPHO1 was initially identified, cloned, and sequenced in chicken cartilage cells as a haloacid dehalogenase (HAD) superfamily member (Houston et al., 1999). Previous research on PHOSPHO1 mainly concentrated on bone mineralization (Dillon et al., 2019). Recent studies have revealed PHOSPHO1 regulated energy metabolism. Specifically, ablation of *PHOSPHO1* could improve glucose tolerance and insulin sensitivity, ameliorate metabolic associated fatty liver disorder (MAFLD), regulate stress-related energy metabolism during erythropoiesis, and stimulate brown adipose tissue (BAT) thermogenesis (Huang et al., 2018; Jiang et al., 2020; Suchacki et al., 2020). We review the structure, regulation and role of PHOSPHO1 and its associated phospholipids homeostasis in metabolic disorders and discuss the possibility of targeting PHOSPHO1 and its regulated phospholipids for treating metabolic disorders in mammalian system.

### Conservation of the *PHOSPHO1* gene

**FIGURE 1:**
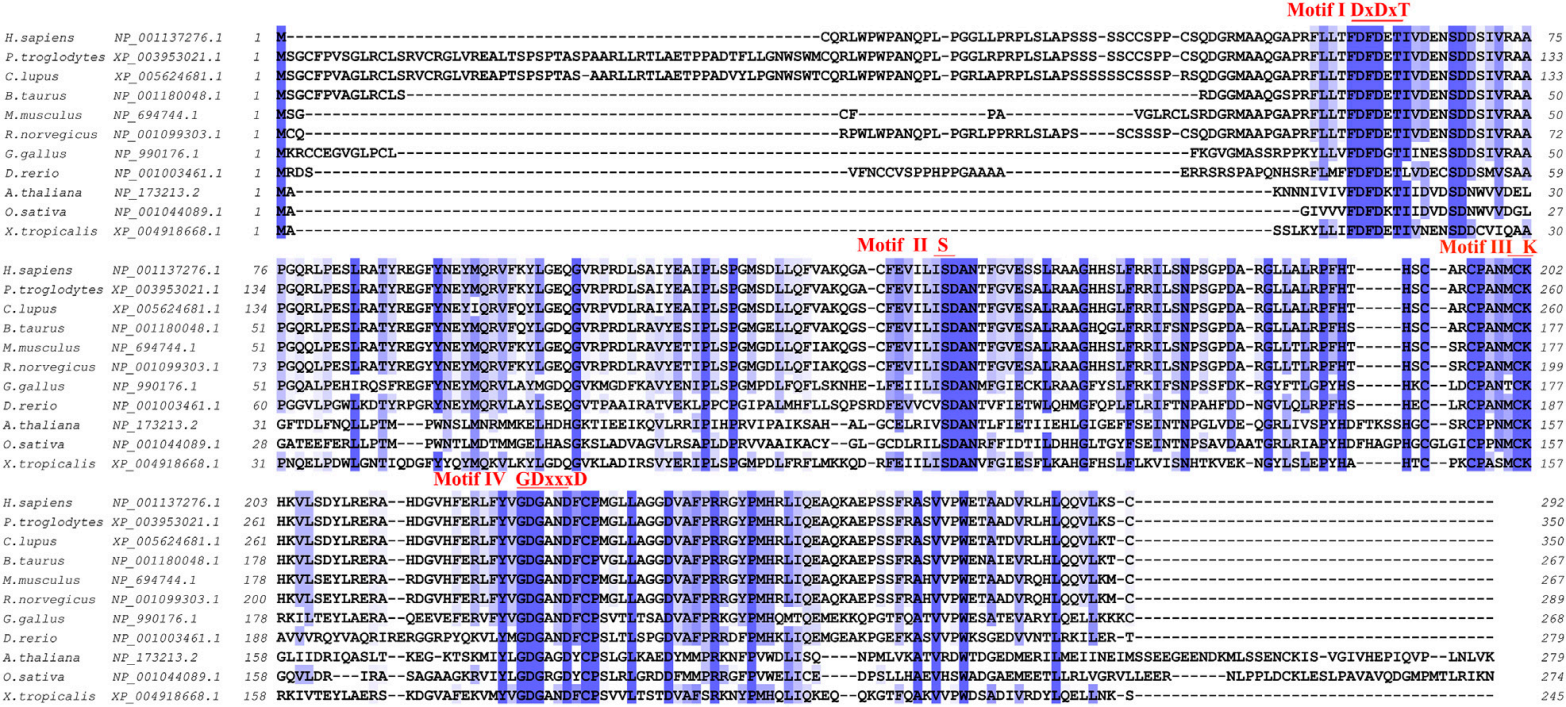
Cross-species amino acid sequence comparisons of PHOSPHO1 orthologs at the four conserved motifs. Alignment was obtained and adapted from homoloGene, T-Coffee and Jalview results (Notredame et al., 2000; Troshin et al., 2011).

*PHOSPHO1* is a highly conservative gene with homologs across multiple species. Human-derived, chicken-derived, and mouse-derived *PHOSPHO1* genes showed conserved synteny, indicating they have the same evolutionary ancestor and are direct homologs (Houston et al., 2002). Comparing the amino acid sequences of PHOSPHO1 from different species, including human, pufferfish, *drosophila*, mouse, rat, chicken, zebrafish and plants, revealed that they all possess the conserved motifs of the HAD superfamily, further demonstrating the homology and ancient evolutionary origin of PHOSPHO1 across multiple species (Stewart et al., 2003) (Figure 1). The HAD superfamily is a pervasive enzyme superfamily founded in a variety of organisms such as *Homo sapiens* and *Arabidopsis thaliana*. This superfamily is functionally classified into phosphatases, ATPases and many other enzyme types (Burroughs et al., 2006; Allen and Dunaway-Mariano, 2009). HAD had four conserved motifs (Figure 1) that play a critical role in the coordination of Mg^2+^ for enzyme catalysis and stabilization of the negatively charged reaction intermediate (Seifried et al., 2013).

### The deoxyribonucleic acid and protein structures of PHOSPHO1

The *PHOSPHO1* gene of humans and mice has three exons (Houston et al., 2002), of which exon 3 contains the conservative motif of the HAD superfamily. A novel spliced *PHOSPHO1* transcript-*PHOSPHO1*-3a-has been identified from human osteoblasts. The 127 bp sequence in intron 2 of *PHOSPHO1*-3a forms the starting point of an 879 bp open reading frame with a predicted protein, which encodes 292 amino acids (Roberts et al., 2008).

**FIGURE 2:**
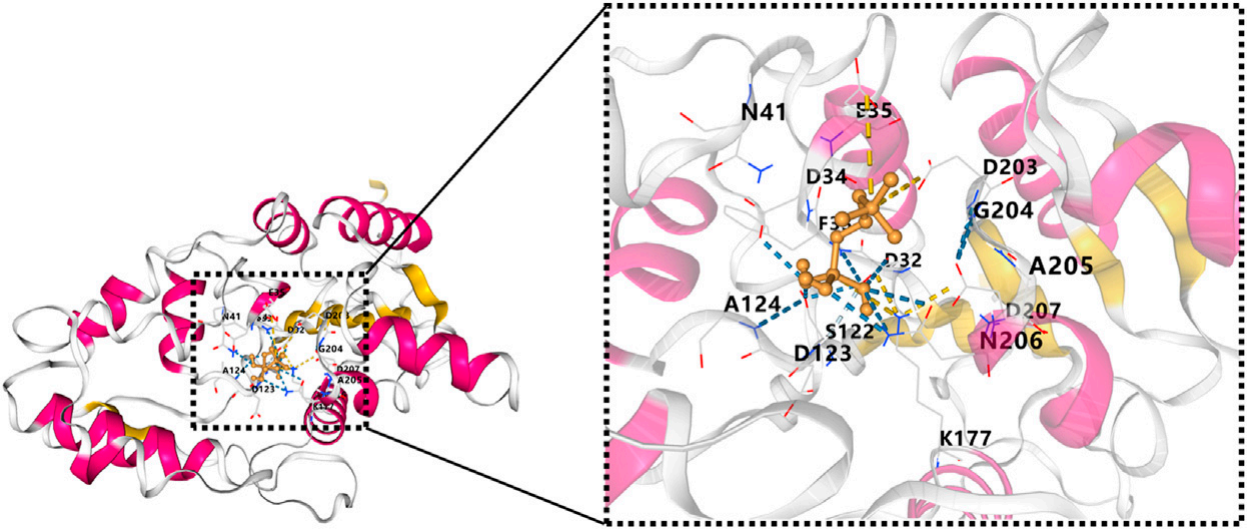
Molecule docking result of AlphaFold predicted 3D structure of mouse PHOSPHO1 protein with phosphocholine by CB-Dock (Liu et al., 2020). The pink color represents α-helices, and the yellow color represents β-sheets.

The InterPro database predicted the HAD domain to be the functional domain of the PHOSPHO1 protein (Mitchell et al., 2015). The crystal structure of PHOSPHO1 protein has not been reported. Researchers constructed the tridimensional model of the human PHOSPHO1 protein based on the phosphoserine phosphatase (PSP) from Methanococcus jannaschii (Wang et al., 2001). The constructed human PHOSPHO1 protein consisted of two domains, including catalysis-related α/β domains and a Rossmann-like fold with a four-spiral bundle domain. The Rossmann-like fold had six parallelisms β-sheets structure consisting of six α-helices surroundings. The substrate Mg^2+^ ion and Asp32, Asp34, and the Asp203 residues interact with octahedral geometry (Stewart et al., 2003). The predicted human PHOSPHO1 protein contained three conserved peptide motifs. The motif I comprised Thr and Val residues and two aspartic acids (Asp43 and Asp123) residues. Mutation of Asp123 reduced the catalytic activity of PHOSPHO1 with phosphoethanolamine and phosphocholine by 20 and 60 times, respectively. Mutation of Asp43 reduced the catalytic activity of PHOSPHO1 with phosphoethanolamine and abolished the reactivity of PHOSPHO1 with phosphocholine. These results indicated that Asp123 and Asp43 of motif I might be the active enzymatic sites of PHOSPHO1 protein in catalyzing different substrates (Stewart et al., 2003). AlphaFold platform developed by DeepMind (Jumper et al., 2021) predicted the 3D structure of mouse PHOSPHO1 protein based on its amino acid sequence. The predicted PHOSPHO1 protein contained a Roseman folding structure comprising five parallel β-sheet structures surrounded by six α-helix structures and additional two β-Sheets and four α-helices. Molecular docking revealed that the substrate phosphocholine would bind to Asp123, Asp32, Asp34, and Asp203 residues of the AlphaFold predicted PHOSPHO1 protein (Figure 2). Human PHOSPHO1 protein is likely located in the cytosol according to the COMPARTMENTS database (Binder et al., 2014). It would be interesting to investigate whether and how PHOSPHO1 substrates are transported or diffused to where PHOSPHO1 is located for efficient catalysis. Human PHOSHO1 is present in matrix vesicles (Dillon et al., 2019). Although there is no direct evidence demonstrating the content of PHOSPHO1 substrates in matrix vesicles, the fact that inhibition of PHOSPHO1 decreased the mineralization capacity of matrix vesicles (Roberts et al., 2007) suggested the importance of PHOSPHO1-mediated enzymatic reactions in matrix mineralization. Similarly, one of the plant homologs of PHOSPHO1-OsACP1-is located in ER and Golgi apparatus. Overexpression or mutation of *OsACP1* altered Pi recycling and plant growth under Pi stress (Deng et al., 2022).

### Regulatory mechanisms of the *PHOSPHO1* gene

Recent studies revealed the transcriptional factor regulation and histone modifications of the *PHOSPHO1* gene. ChIP-seq experiments illustrated that C/EBPβ and PPARγ bind to PHOSPHO1 promoter in mouse embryonic fibroblasts C3H10T1/2 cell (Lachmann et al., 2010). PPARγ and C/EBP are critical factors regulating adipogenesis (Madsen et al., 2014). The ChIP-seq result suggested that PHOSPHO1 may be involved in PPARγ- and C/EBP-regulated adipose development. The ENCODE Histone Modification Site Profiles dataset showed histone modifications, such as H3K27ac, H3K4me1, and H3K4me3, in an intron region of the *PHOSPHO1* gene in mouse brown adipose tissue. These histone modifications usually indicated an active enhancer or promoter (Giddings et al., 2011), suggesting active expression of *PHOSPHO1* in BAT.

**FIGURE 3:**
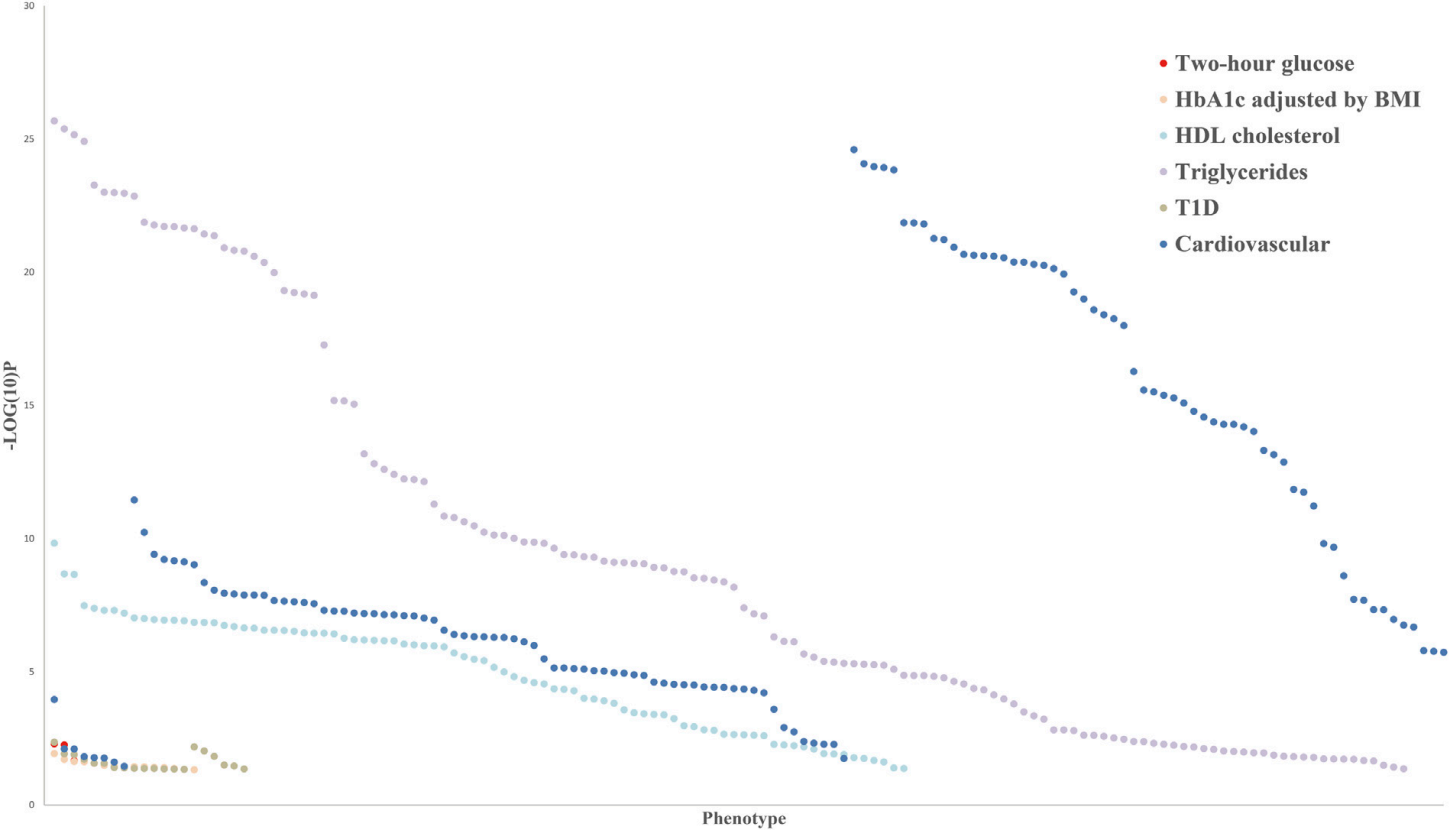
Common variant gene-level associations for PHOSPHO1 as the closest gene with metabolic disorders indicators.

Epigenomic association studies indicated that the methylation levels of *PHOSPHO1* in whole blood of subjects were positively correlated with their HDL cholesterol levels (Dayeh et al., 2016; Sayols-Baixeras et al., 2016) and negatively correlated with the future risk of developing type II diabetes (T2D) (Chambers et al., 2015). DNA methylation of PHOSPHO1 locus cg02650017 was decreased in skeletal muscle of diabetic patients compared with their non-diabetic twin siblings (Dayeh et al., 2016). The SNP of *PHOSPHO1* from whole blood samples has been reported to be correlated with the body mass index (BMI), blood pressure, and waist-to-hip ratio of subjects by a genome-wide association study (Wu et al., 2018; Li et al., 2021). Additionally, the SNP of *PHOSPHO1* is associated with the level of sex hormone-binding globulin (Sinnott-Armstrong et al., 2021), a predicting factor of T2D risk (Wang et al., 2015). Blood eQTL analysis revealed that *PHOSPHO1*, which generates inorganic Pi and contributes to vascular calcification, was associated with the development of cardiovascular disease (Kiffer-Moreira et al., 2013; Bobryshev et al., 2014; Leblanc et al., 2016). According to human genetics knowledge portals, there are *PHOSPHO1* related variants, including missense mutations, intron variants, transcription factor binding site variants and 3′-UTR variants, which are associated with metabolic phenotypes, such as cardiovascular disease, diabetes and glucose and lipid metabolism (Figure 3). Those genetic variants may alter gene expression and protein activity by influencing transcription, epigenetic modifications, and the binding of microRNAs and RNA-binding proteins (Steri et al., 2018; Petrosino et al., 2021; Tseng et al., 2021). The exact mechanisms by which the *PHOSPHO1* variants influence the expression or activity of *PHOSPHO1* and its associated metabolic phenotypes await future genetic studies for clarification.

The expression of *PHOSPHO1* was related to metabolic disorders. In addition to reported expression in skeletal tissue, *PHOSPHO1* is highly enriched in both mice and human BAT, the thermogenic adipose tissue. *PHOSPHO1* is co-expressed with several mitochondrial genes and may participate in mitochondrial electron transport and fatty acid metabolism (Jiang et al., 2020). *PHOSPHO1* transcript was increased in the liver of nonalcoholic steatohepatitis patients (Liu et al., 2011) and diminished in the liver of hepatitis mice, myocardial tissue of mice with left ventricular hypertrophy (Mirotsou et al., 2006), and muscle of mice with type 1 diabetes mellitus (Lehti et al., 2006; Barrett et al., 2013). AAV-mediated Fibroblast growth factor 21 (*FGF21*) gene therapy increased *PHOSPHO1* expression in iWAT of the high-fat diet (HFD)-fed mice (Jimenez et al., 2018). Nonetheless, more evidence is needed to determine whether the altered expression of *PHOSPHO1* is directly involved in metabolic disorders or a reflection of the regulatory feedback response.

The InterPro database defines *OsACP1*, *PPsPase1* and *PECP1* as phosphatases related to PHOSPHO1/2 of HAD superfamily in plants (Hunter et al., 2009). Phosphate starvation in plants induced the expression of *OsACP1* and *PECP1*, which in turn either maintains Pi homeostasis (Deng et al., 2022) or hinders root architecture response of Pi starvation (Tannert et al., 2018). There has been no direct evidence of *PHOSPHO1* regulation by Pi in the mammalian system. The paralleled induction of *PHOSPHO1* expression and Pi concentrations in osteoblasts, chondrocytes, and odontoblasts during matrix mineralization suggests an association between the two, which warrants further experimental investigation to determine the causal relationships.

### The function of PHOSPHO1 in energy metabolism

In response to exercise and cold exposure, white adipocytes could be transformed into beige adipocytes, which are morphologically and functionally similar to brown adipocytes (Cheng et al., 2021). Knockout of *UCP1*, the thermogenic driver, increased the mRNA and protein expression of *PHOSPHO1* in adipose tissues of cold exposed mice. Knockdown of *PHOSPHO1* by siRNA augmented the expression of *UCP1* in clonal human brown adipocytes. This compensatory regulation suggested that *PHOSPHO1* participate in UCP1-independent adipocyte respiration (Kazak et al., 2015). *PHOSPHO1* ablation induced the expression of thermogenic genes and mitochondria-related genes in BAT, subcutaneous white adipose tissue in mice, and mouse primary brown adipocytes, enhancing cold tolerance and energy expenditure. In addition, depletion of the *PHOSPHO1* gene ameliorated HFD-induced obesity, MAFLD, and insulin resistance in mice, indicating that PHOSPHO1 negatively regulates BAT activation and energy metabolism (Gliniak and Scherer, 2020; Jiang et al., 2020). Understanding the mechanisms that negatively regulate BAT activation is crucial to preventing excessive adipose thermogenesis and overheating and achieving the fine-tuned regulation of energy homeostasis. Cold exposure increased total phosphatidylcholine (PC) and phosphatidylethanolamine (PE) content in mouse BAT (Marcher et al., 2015; Lynes et al., 2018; Sanchez-Gurmaches et al., 2018; Pernes et al., 2021), and dynamically regulated total PC and PE content in mouse WAT (Lynes et al., 2018; Xu et al., 2019; Pernes et al., 2021). The extent of changes in PE and PC fractions was not only greater, but also lasted longer in BAT than in WAT by cold stimulation (Lu et al., 2017). Since *PHOSPHO1* expression was induced by cold exposure in BAT of mice (Jiang et al., 2020), it would be interesting to investigate whether PHOSPHO1 regulated thermogenesis is mediated by alterations in adipose PC and PE contents. Another study revealed that PHOSPHO1 was a regulator of insulin resistance and obesity. Mice lacking *PHOSPHO1* showed improved basal glucose homeostasis and were protected from HFD-induced obesity and diabetes, which was independent of altered bone secreted factors. Choline supplementation restored insulin sensitivity and adiposity in *PHOSPHO1* knockout mice (Suchacki et al., 2020). Consequently, inhibition of PHOSPHO1 activity could potentially treat obesity and related metabolic disorders. The phenotypes resulting from overexpression of *PHOSPHO1* have not been reported yet, which would be important to complete our understanding of PHOSPHO1’s function in metabolic disorders.

The phosphocholine metabolism and *PHOSPHO1* expression were increased during terminal erythropoiesis. The number of erythrocytes and mean corpuscular volume levels were normal in seven to twelve-week-old *PHOSPHO1* knockout mice. In the phenylhydrazine-induced hemolytic anemia model, *PHOSPHO1* knockout mice exhibited defects in stress erythropoiesis, and switched to glycolysis for compensatory energy supply (Huang et al., 2018). These results indicated a role for PHOSPHO1 in stress-related energy metabolism. The expression of *PHOSPHO1* was increased in blood samples from athletes with high-altitude training and vigorous intensity exercise (Glotov et al., 2022). Given that hypoxia causes stress erythropoiesis (Wang et al., 2021), Whether PHOSPHO1 regulates energy metabolism in high-altitude adaptation-induced erythropoiesis remains to be explored.

The increased expression of *PHOSPHO1* during brown adipocytes, erythrocyte differentiation, and calcification were accompanied by decreased content of PC or PE (Wu et al., 2002; Huang et al., 2018; Jiang et al., 2020). Although it was unclear whether changes in PC and PE contents resulted from induction of PHOSPHO1, this evidence suggested that PHOSPHO1 regulated phospholipid homeostasis may not be limited to changes in phosphocholine and phosphoethanolamine in the mammalian system.

Oxidized phospholipid (OxPL) was generated by free radicals attacking and oxidizing phospholipids, which promotes the development of atherosclerosis, nonalcoholic steatohepatitis (NASH) and T2D (Nagashima et al., 2002; Lee et al., 2012; Tangvarasittichai, 2015; Sun et al., 2020). UCP1-mediated proton leakage and thermogenesis have been reported to reduce mitochondrial superoxide production (Brand, 2000; Oelkrug et al., 2010). Since *PHOSPHO1* ablation induced the expression of thermogenic genes and promoted thermogenesis, ablation of *PHOSPHO1* could presumably decrease superoxide and OxPL generation, which may reduce the risk of OxPL-associated cardiovascular and other metabolic diseases.

### Potential role of PHOSPHO1 inhibitors in treating metabolic disorders

Based on the role of PHOSPHO1 in regulating energy metabolism, PHOSPHO1 inhibitors could be developed to treat metabolic disorders. A panel of PHOSPHO1 inhibitors was screened from chemical libraries (Roberts et al., 2007; Kiffer-Moreira et al., 2013; Bravo et al., 2014), among which several proton pump inhibitors were reported to enhance the effect of antidiabetic medications in animal models or T2D patients (Mefford and Wade, 2009; Hove et al., 2010; Swamy et al., 2010; Boj-Carceller et al., 2011; Barchetta et al., 2015; Bozkuş et al., 2020; Gamil et al., 2020). These studies suggested that proton pump inhibitors could serve as adjunctive therapy for T2D. The gavage of lansoprazole alone also significantly decreased body weight and fat mass in HFD-fed mice (Benchamana et al., 2019). Ebselen, another PHOSPHO1 inhibitor (Roberts et al., 2007), could improve insulin sensitivity (Wang et al., 2014; Polianskyte-Prause et al., 2022) and ameliorate diabetes-associated atherosclerosis (Chew et al., 2010) in mice. The mechanisms underlying proton pump inhibitors-induced metabolic benefits remain unclear. Based on the inhibition of PHOSPHO1 activity by proton pump inhibitors and the effect of PHOSPHO1 deletion on glucose tolerance and insulin resistance, PHOSPHO1 may be involved in proton pump inhibitors-regulated metabolic homeostasis, which requires further experimental validation in the future.

Although PHOSPHO1-mediated bone mineralization systems were crucial for ossification, there was no evident proof that PHOSPHO1 affects the grown-up skeleton. The bone matrix mineralization indexes, such as strain and stiffness, would correct with age in *PHOSPHO1* knockout mice (Javaheri et al., 2015). The use of reported PHOSPHO1 inhibitors did not impair bone regeneration or influence bone mineralization in murine models (Dillon et al., 2019; Gul et al., 2022), so PHOSPHO1 inhibitors may regulate energy metabolism without causing skeleton impairment, at least in adulthood. Similarly, hemolytic anemia was not reported in adult *PHOSPHO1* knockout mice. Moreover, red blood cell properties, such as number, morphology and osmotic fragility, were not changed in *PHOSPHO1* knockout mice (Huang et al., 2018). These data suggested that PHOSPHO1 inhibitors should not impair normal erythropoiesis. Accordingly, future functional experiments can be carried out with screened PHOSPHO1 inhibitors to evaluate the safety and effectiveness of PHOSPHO1 inhibitors in treating metabolic disorders.

## PHOSPHO1-regulated phospholipid metabolism in mammalian metabolic disorders

### Metabolism and function of phosphocholine

PHOSPHO1 catalyzes the hydrolysis of phosphoethanolamine and phosphocholine to generate choline and ethanolamine, respectively, thereby affecting phospholipid metabolic homeostasis. This reaction involves the Kennedy pathway. In the CDP-choline pathway, choline is catalyzed to phosphocholine by choline kinase, which is converted to CDP-choline via CTP-phosphocholine cytidylyltransferase. CDP-choline produces PC by 1, 2-diacylglycerol choline phosphotransferase (Gibellini and Smith, 2010; Onono and Morris, 2020).

Phosphocholine and its related metabolite levels are closely associated with energy metabolism. The addition of serum into cell culture increased choline kinase activity, promoting phosphocholine and PC synthesis in 3T3 cells (Warden and Friedkin, 1985). Human plasma metabolomics studies demonstrated that plasma phosphocholine was negatively correlated with BMI and HOMA-IR of subjects (Palomino-Schätzlein et al., 2020), suggesting that low levels of plasma phosphocholine could be used as a metabolic biomarker for insulin resistance. Comparative metabolomics showed that the level of phosphocholine in BAT was less than those in subcutaneous white adipose tissue of mice. Phosphocholine content in BAT was increased in response to cold stimulation and β-adrenergic receptor agonism (Mills et al., 2018), suggesting the regulation of β-adrenergic signaling on phosphocholine homeostasis. The increased level of phosphocholine could be the result of enhanced production or reduced degradation of phosphocholine, which warrants further investigations to confirm.

**FIGURE 4:**
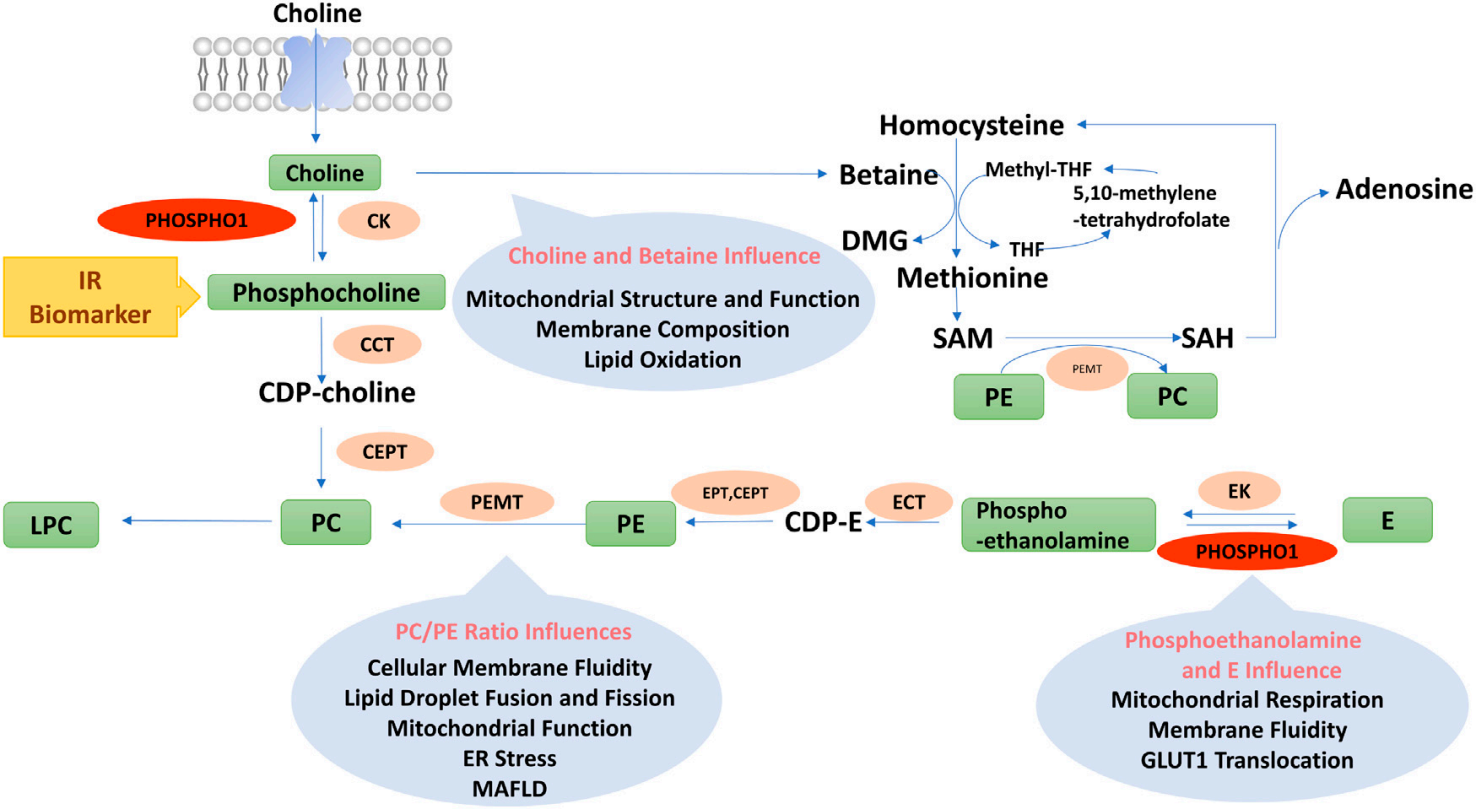
Diagram of phospholipids metabolism pathways. PC, Phosphatidylcholine; PE, Phosphatidylethanolamine; CDP-E, CDP-ethanolamine; E, Ethanolamine; DMG, Dimethylglycine; Methyl-THF, 5-methyltetrahydrofolate; THF, Tetrahydrofolate; SAM, Adenosylmethionine; SAH, Adenosylhomocysteine; LPC, Lyso-phosphatidylcholine; PEMT, Phosphatidylethanolamine n-methyltransferase; E/CK, Ethanolamine/choline kinase; E/CCT, Phosphoethanolamine/phosphocholine cytidylyltransferase; EPT, Ethanolamine phosphotransferase; CEPT, Choline/ethanolamine phosphotransferase.

Endometrial cancer was correlated with a diversity of metabolic disorders, such as obesity, hypertension, and diabetes (Kyo and Nakayama, 2020). The increased level of phosphocholine is the metabolic character of the well-differentiated and low-grade endometrial cancer type (Skorupa et al., 2021). The ratio of o-phosphocholine to UDP-N-acetylglucosamine within pancreatic β-cells is a probable indicator of glucotoxicity, lipotoxicity, glucolipotoxicity, and metabolic imbalances associated with T2D (Yousf et al., 2019). Consequently, drug intervention or lifestyle changes could be applied based on biomarkers changes as early as possible to reduce the future risk of metabolic disorders (Figure 4).

### Metabolism and function of choline

Choline is catalyzed by choline kinase to produce phosphocholine in an ATP- and Mg^2+^-dependent manner. There are two isoforms of choline kinase: CHKα and CHKβ. CHKβ mutations cause rostrocaudal muscular dystrophy (Sher et al., 2006). Mice lacking *CHKβ* had significantly decreased muscle PC content and impaired mitochondrial production, as evidenced by considerably reduced mitochondrial respiratory complex activity, decreased ATP synthesis, increased oxidative stress and activation of mitochondrial autophagy in the fore and hind limb muscles (Chen et al., 2017). Except for mouse models, clinical subjects with congenital muscular dystrophy bore *CHKβ* mutations with reduced PC levels in their skeletal muscle (Mitsuhashi et al., 2011). PC is a vital component of the mitochondrial membrane structure by facilitating the assembly of the outer mitochondrial membrane translocase TOM complex and the biogenesis of β-barrel proteins (Bohnert et al., 2012; Schuler et al., 2015). Consequently, *CHKβ* deficiency leads to decreased production of phosphocholine and PC, which in turn impairs mitochondrial structure and function.

Choline, a vital nutrient of the body, can produce membrane phospholipids and acetylcholine or generate methyl donors, such as betaine and S-adenosylmethionine. Choline is transported into cells by the choline transporter. Hindering choline uptake by macrophages attenuated the activation of NLRP3 inflammasome and the production of proinflammatory cytokines (Sanchez-Lopez et al., 2019). Decreased choline uptake altered mitochondrial membrane composition, inhibited mitochondrial ATP synthesis, and enhanced mitochondrial autophagy, resulting in inflammation suppression (Sanchez-Lopez et al., 2019). By activating AMP-activated protein kinase (AMPK), choline phosphorylated and inactivated acetyl coenzyme A carboxylase (ACC), then reduced intracellular malonyl coenzyme A and fatty acid synthesis in the liver (Lee M et al., 2018). PPARα, the main regulator of mitochondrial fatty acid metabolism, was activated by choline, which increased fatty acid oxidation (Schenkel et al., 2015). Altering exogenous choline consumption affected body weight. The choline-deficient diet reduced body weight and body fat gain, decreased blood glucose levels, and improved insulin sensitivity in the leptin-deficient *ob/ob* mice (Wu et al., 2012). Clinical trials reported that serum choline levels from fasted subjects were inversely associated with the patient’s body weight or BMI (Kochhar et al., 2006; Chen et al., 2015). The results of animal experiments and clinical trials contradicted, possibly because animal experiments could strictly restrict choline intake through diet, while it was challenging to limit choline intake in subjects of clinical trials completely. In addition, the serum choline levels may not accurately reflect dietary choline intake (Gao et al., 2016). Gender also affects the relationship between choline and body composition. Serum choline was inversely and positively associated with BMI and body fat in male and female subjects, respectively (Gao et al., 2018). As a result, the relationship between circulating choline level and body composition needs to be discussed within the context of a specific gender, fasting state, or dietary conditions.

Choline might also influence metabolic functions by regulating host intestinal microbiota. Chronic HFD feeding altered the physiological structure of the intestinal epithelium, which increased the ability of *Escherichia coli* to break down choline. Choline is converted to trimethylamine and trimethylamine N-oxide by intestinal microorganisms and hepatic drug-metabolizing enzymes (Yoo et al., 2021). Trimethylamine N-oxide binds to and activates the protein kinase R-like endoplasmic reticulum kinase (PERK), which causes endoplasmic reticulum (ER) stress and phosphorylates the transcription factor FOXO1 (Chen et al., 2019), leading to insulin resistance (Zhang et al., 2013). Therefore, choline and its metabolites play a complex role in metabolic disorders by affecting mitochondrial function, inflammation, and microbiota function (Figure 4).

### Metabolism and function of ethanolamine and phosphoethanolamine

Ethanolamine and phosphoethanolamine could be interconverted via ethanolamine kinase and PHOSPHO1. Ethanolamine, a rich component of bacterial cell membranes, plays an integral role in dietary lipid nutrients (Zhou et al., 2017). Ethanolamine was considered a carbon and nitrogen source to influence the number and ratio of colonic microorganisms (Patel and Witt, 2017), affecting the homeostasis of host metabolism (Zhou et al., 2018).

The cellular membrane fluidity influenced glucose transporter 1 (GLUT1) translocation to the cell membrane (Perona, 2017). Ethanolamine treatment increased its conversion to phosphatidylethanolamine (PE), which decreased cellular membrane fluidity, impairing GLUT1 translocation and glucose uptake in tumor cells (Garlapati et al., 2021). The altered membrane fluidity also influenced mitochondrial structure and function. As a result of impaired glucose utilization and mitochondrial dysfunction, tumor cells, otherwise dependent on glucose function, increase lipolysis as an alternative energy source (Garlapati et al., 2021). In addition, several studies have confirmed that phosphoethanolamine inhibits mitochondrial respiration (Modica-Napolitano and Renshaw, 2004). Succinate was an endogenous substrate of mitochondrial complex II, and phosphoethanolamine negatively controlled mitochondrial activity by directly competing with succinate in mitochondrial complex II (succinate dehydrogenase) (Fontana et al., 2020). The above findings indicated the important role of phosphoethanolamine and ethanolamine in regulating intestinal microbiome, membrane fluidity, mitochondrial function, and energy metabolism (Figure 4).

### Metabolism and function of betaine

Betaine, also known as trimethylglycine, is metabolized from choline. Betaine is a vital methyl donor, which generates dimethylglycine (DMG) catalyzed by betaine-homocysteine S-methyltransferase (BHMT). Homocysteine (Hcy) receives a methyl group from betaine and generates l-methionine and DMG (Zhao et al., 2018). Methionine and ATP generated S-adenosylmethionine (SAMs) in the presence of methionine adenosyltransferase. SAM is catalyzed to S-adenosylhomocysteine (SAH) by methyltransferase. SAH is converted to Hcy by the action of S-adenosyl homocysteine hydrolase (SAHH), which is then converted to cysteine and the antioxidant glutathione by vitamin B6 and cystathionine-β-synthase (CBS) (Cavallaro et al., 2017). Thus, betaine inhibited superoxide-induced free radical generation and had anti-inflammatory functions by inhibiting the NF-κB pathway (Go et al., 2005) and NLRP3 inflammasome activation (Kim et al., 2017). Betaine also ameliorated hepatocyte insulin resistance by enhancing tyrosine phosphorylation of IRS-1 and thus activating PKB/AKT signaling (Kathirvel et al., 2010).

Betaine supplementation reduced hepatic lipid accumulation, subcutaneous and visceral white fat mass, improved insulin sensitivity and increased white adipose tissue mitochondrial content in HFD-fed mice (Wang et al., 2010; Du et al., 2018). Betaine enhanced fatty acid oxidation and inhibited fatty acid synthesis by increasing polyunsaturated fatty acids as ligands to activate PPARα and decreasing sterol regulatory element-binding protein-1c (SREBP-1c) expression (Echeverría et al., 2016), which in turn reduced intracellular lipid accumulation in HFD-fed mice. In addition, betaine activated AMPK by generating SAM that could directly bind to the CBS structural domain of AMPK. On the other hand, SAM was converted to SAH, which produced Hcy and AMP, leading to an enhancement in the AMP/ATP ratio and thus activating AMPK and the catabolic pathways (Carling, 2017) (Figure 4).

### Metabolism and function of phosphatidylserine

PC consists of 95% of the total choline pool in animals (Li and Vance, 2008). The inner lipid bilayer of the cell membrane of most eukaryotic cells consists of phosphatidylserine (PS) and PE, while the outer lipid bilayer consists of PC and sphingolipids (SM) (Hafez and Cullis, 2001). In addition, PE could be converted to PC by phosphatidylethanolamine N-methyltransferase (PEMT).

Several studies have demonstrated that PC was involved in regulating energy metabolism. Cellular experiments had shown that inhibition of PC synthesis affected ER morphology and was accompanied by impaired protein transport in the Golgi complex (Testerink et al., 2009). PC was the major phospholipid component on the surface of lipoproteins such as VLDL and LDL. Therefore, impaired hepatic PC biosynthesis significantly reduced circulating VLDL levels and alleviated hyperlipidemia, but caused MAFLD due to VLDL reduction (Van Der Veen et al., 2017; Packard and Taskinen, 2020). As an adipose tissue immune cell population, macrophages were involved in insulin resistance during obesity development. Adipose tissue macrophages produced PC in large quantities by activating phosphocholine cytidylyltransferase α (CCTα). The reduced PC conversion in macrophages from mice with macrophage-specific knockout of *CCTα* increased the half-life of PC, which was able to incorporate more polyunsaturated fatty acids, reduced ER stress, and mitigated adipose tissue inflammation and insulin resistance (Robblee et al., 2016; Petkevicius et al., 2019).

Clinical studies have discovered that the levels of PC and its derivatives were associated with energy metabolism. Serum levels of diacyl-phosphatidylcholine C36:1, C38:3 and C40:5 in 27,548 participants were associated with their future risk of T2D (Floegel et al., 2013). The serum level of diacyl-phosphatidylcholine C32:1 was positively associated with healthy metabolic parameters, whereas those of diacyl-phosphatidylcholine C32:2 and C34:2 were positively associated with unhealthy metabolic parameters in obese subjects (Bagheri et al., 2018; Blüher, 2020). A serum metabolomics study reported a positive connection between BAT activity and serum acyl-lysophosphatidylcholine levels in male subjects (Boon et al., 2017). Lower fasting serum lysophosphatidylcholine levels could predict impaired glucose tolerance as well as the hazard of developing T2D (Wang-Sattler et al., 2012). Therefore, PC-regulated phospholipid components could impact ER function and insulin sensitivity, further influencing the development of metabolic disorders (Figure 4).

### Metabolism and function of phosphatidylethanolamine

PE, an important component of the cell membrane, is synthesized through the CDP-ethanolamine Kennedy pathway (Gibellini and Smith, 2010). Ethanolamine is phosphorylated to produce phosphoethanolamine via ethanolamine kinase (EK). The second step of the Kennedy pathway is the rate-limiting step, in which CTP-phospholipid amide cell transferase (Pcyt2) transferred CTP to phosphoethanolamine to form CDP-ethanolamine. Finally, CDP-ethanolamine is catalyzed by 1, 2-diacylglycerol ethanolamine-phosphotransferase (EPT) to condense with diacylglycerol to produce PE. Pcyt2 is the rate-limiting enzyme for PE synthesis (Bakovic et al., 2007). In addition, phosphatidylserine decarboxylase (PISD) can decarboxylate PS that is taken from the ER to the mitochondria to produce PE (Vance, 1990).

*Pcyt2* gene is essential for embryo development, whose deletion could cause embryonic lethality (Singh et al., 2012). Although the rate of PE biosynthesis is decreased in *Pcyt2* ^+/−^mice, a single *Pcyt2* allele maintains phospholipid homeostasis. Thus, young *Pcyt2* ^+/−^ mice were asymptomatic. However, the reduced CDP-ethanolamine in *Pcyt2* ^+/−^ mice prevented the efficient synthesis of PE from ethanolamine and diglycerides, increasing triglyceride synthesis and inhibiting fatty acid oxidation (Fullerton et al., 2009). Thus, aged *Pcyt2* ^+/−^mice gradually developed defects in fatty acid metabolism, which led to obesity, MAFLD, and insulin resistance (Grapentine et al., 2022). Supplementation with PE reversed the MAFLD and hepatic inflammation in *Pcyt2* ^+/−^ mice (Grapentine et al., 2022). The addition of choline to drinking water reduced triglyceride synthesis, elevated fatty acid oxidation, increased muscle glycogen stores, and restored insulin sensitivity in skeletal muscle of *Pcyt2* ^+/−^ mice. Choline treatment inhibited mTOR phosphorylation by activating AMPK and AKT, thereby restoring muscle glucose metabolism in insulin-resistant *Pcyt2* ^+/−^ mice (Taylor et al., 2017). These outcomes suggested an essential role of the Pcyt2 and CDP-ethanolamine Kennedy pathway in the progression of MAFLD (Figure 4).

### The balanced ratio of phosphatidylserine and phosphatidylethanolamine plays an essential role in energy metabolism

In addition to their absolute levels, the balanced ratio of PC and PE also plays a critical role in energy metabolism. PC and PE function differently as membrane structural components. PC tends to form cylindrical molecules that are polymerized into mobile lipid bilayers with connected tails and hydrophilic polar head groups, whereas PE forms conical molecules that increase membrane curvature and affect membrane outgrowth, division, fusion, and membrane protein embedding (Van Meer and De Kroon, 2011). Thus, the ratio of PC/PE influenced ER homeostasis, mitochondrial function, and lipid droplet fusion and fission.

PEMT converts PE to PC. *PEMT* knockout mice exhibited elevated oxygen consumption rates, reduced hepatic gluconeogenesis, and suppressed HFD-induced obesity and insulin resistance. However, *PEMT* ablation decreased the PC/PE ratio and reduced the synthesis and secretion of VLDL, which ultimately caused ER stress and MAFLD (Wan et al., 2019; Gao et al., 2015). Lipidomics studies revealed a higher PC/PE ratio in obese mice than in lean mice. Chronic ER stress was also presented in the obese mice livers. Inhibiting hepatic PEMT expression in obese mice corrected the PC/PE ratio and alleviated ER stress (Fu et al., 2011). The altered PC/PE ratio disrupted membrane lipid homeostasis and resulted in lipid bilayer stress, which activated inositol-requiring enzyme 1 (IER1) to induce ER stress (Gao et al., 2015; Ishiwata-Kimata et al., 2022). Therefore, the amounts of PC and PE need to be maintained in a delicate balance to avoid ER stress.

In addition, the PC/PE ratio impacted mitochondrial functions. PE is synthesized by phosphatidylserine decarboxylase or ethanolamine kinase and is exported from mitochondria to mitochondria-associated membranes and ER, where it is converted to PC by PEMT (Vance, 1990). Inhibition of PEMT in mouse 3T3-L1 mature adipocytes and PEMT knockout mice increased mitochondrial CoQ content (Park et al., 2010) and produced more ATP (Van Der Veen et al., 2014). PEMT deficiency altered the concentration of single-carbon metabolites in mitochondria, elevated the SAM: SAH ratio, and increased mitochondrial methylation capacity and CoQ synthesis, which accelerated respiratory chain electron transport and ATP production (Turunen et al., 2004).

The ratio of PC/PE also influenced lipid droplet fusion and fission. PC works as a surfactant to prevent the fusion of lipid droplets. PE could produce larger lipid droplets because PE destabilizes the lipid droplet membrane and induces lipid droplet fusion (Krahmer et al., 2011; Cohen et al., 2015). The decreased ratio of PC/PE promoted lipid droplet fusion, which reduced the total surface area of lipid droplets in white adipose tissue (Guo et al., 2008). Thus, the small surface area of lipid droplets in white adipose tissue restricted their contact with lipase, resulting in inefficient lipolysis (Nishimoto and Tamori, 2017).

To sum up, the PC/PE ratio is essential in influencing the function of mitochondria and lipid droplets. Although inhibition of PEMT ameliorated insulin resistance in HFD-fed mouse models, it also resulted in MAFLD development due to lower secretion of VLDL. In addition, an increased level of either PC or PE alters the PC/PE ratio, which might cause ER stress. Therefore, inhibiting the production of PC or PE alone is likely to fail to improve the metabolic phenotype, as the ratio of PC/PE needs to be precisely balanced to avoid ER stress and MAFLD development (Figure 4).

### Effects of knocking down phospholipid-related enzymes on lipid droplet parameters in human THP-1 macrophages

**FIGURE 5:**
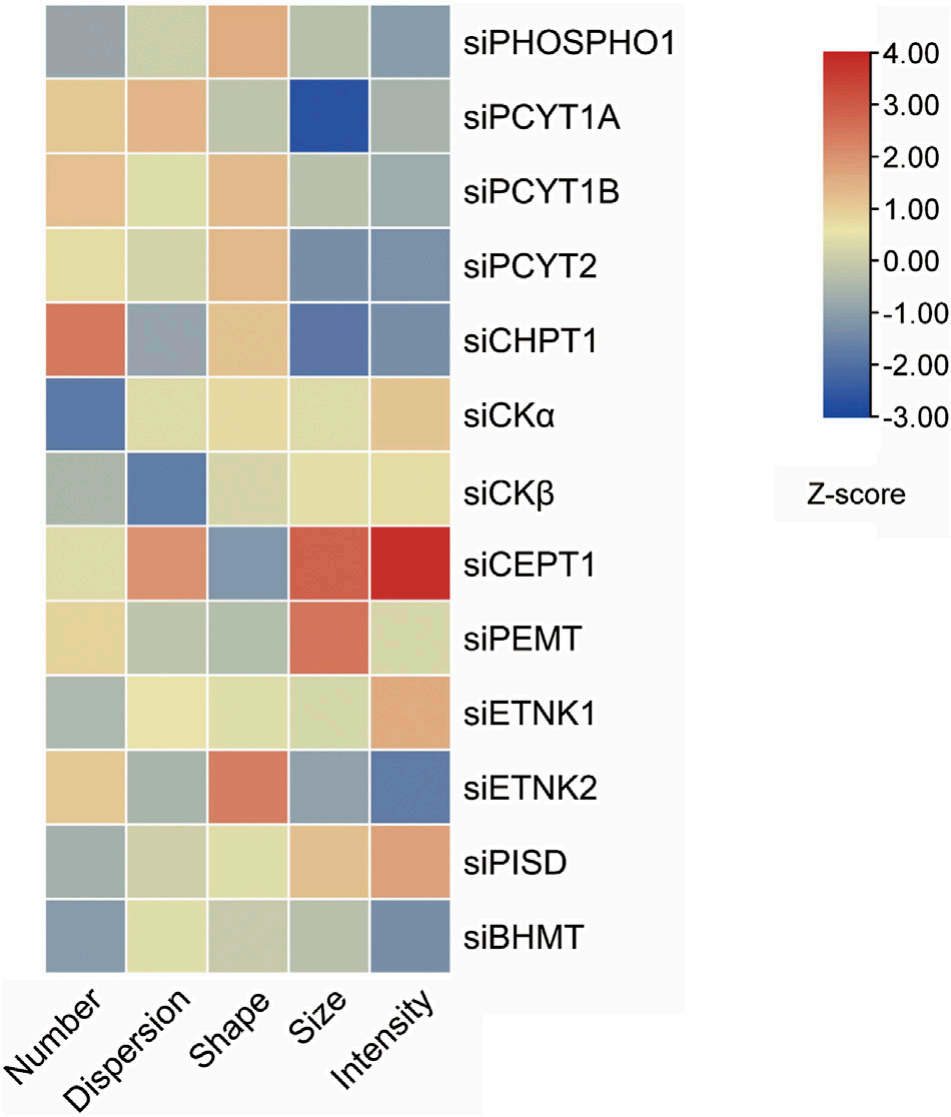
Knockdown of phospholipids-regulating genes in THP-1 macrophages alters lipid droplets morphology induced by oleic acid. Five features of lipid droplets were selected and scaled to generate z-scores (Mejhert et al., 2020) and the heatmap.

Overloading macrophages with lipid droplets is one main reason for insulin resistance (Lee YS et al., 2018). The lipid droplet knowledge portal database collected genes that modify lipid storage in human THP-1 macrophages based on RNAi screen results (Mejhert et al., 2020). We searched the database with phospholipid-regulating genes mentioned in this review. The Z scores of five dimensions, including the number, size, shape, intensity, and dispersion of lipid droplets, of lipid storage from knockdown of phospholipid-regulating genes were present as a heatmap (Figure 5). Knockdown of *PEMT* in THP-1 macrophages resulted in a larger size of lipid droplets when fed with oleic acid compared with those of control cells. The phenotype could be due to *PEMT-*silencing induced PE accumulation, which promoted lipid fusion and resulted in larger lipid droplets.

## Summary and outlook

Previous studies on PHOSPHO1 mainly focused on bone mineralization, while recent studies identified novel functions of PHOSPHO1 and its regulated phospholipid homeostasis on energy metabolism, which in turn serve as potential therapeutic candidates for the metabolic disorders, including obesity, T2D, and MAFLD. This review summarized the structure and upstream regulatory mechanisms of PHOSPHO1, outlined the functions of PHOSPHO1 and its related phospholipid metabolites in metabolic disorders, and examined mechanistic evidence of phospholipid regulation of mitochondrial and lipid droplets in the context of metabolic homeostasis. The recent development of metabolic flux tracing and spatial metabolomics enable monitoring the temporal and spatial phospholipids metabolism pathways to identify key enzymes that contribute to metabolic dysfunctions and provide therapeutic opportunities to restore metabolic functions. Future studies utilizing tissue-specific *PHOSPHO1* knockout and transgenic mouse models could dissect the role of PHOSPHO1 in metabolically active tissues and organs, such as adipose tissue, liver and skeletal muscle. At the same time, it is also necessary to carefully evaluate the effect and mechanism of PHOSPHO1 inhibitors on energy metabolism to provide novel strategies for the prevention and treatment of chronic metabolic disorders that plague human beings in the era of the obesity pandemic.
